# Continuous Vital Signs Monitoring with a Wireless Device on a General Ward: A Survey to Explore Nurses’ Experiences in a Post-Implementation Period

**DOI:** 10.3390/ijerph20105794

**Published:** 2023-05-11

**Authors:** Femke L. Becking-Verhaar, Robin P. H. Verweij, Marjan de Vries, Hester Vermeulen, Harry van Goor, Getty J. Huisman-de Waal

**Affiliations:** 1 Department of Surgery, Radboud University Medical Centre, Huispost 751, Postbus 9101, 6500 HB Nijmegen, The Netherlands; 2 Scientific Institute for Quality of Healthcare, Radboud Institute for Health Sciences, Radboud University Medical Centre, Huispost 160, Postbus 9101, 6500 HB Nijmegen, The Netherlands

**Keywords:** continuous monitoring, early warning system, fundamental care, implementation, nursing, remote monitoring, thematic analysis, vital signs, wearable devices, wireless monitoring

## Abstract

Background: Nurse engagement, perceived need and usefulness affect healthcare technology use, acceptance and improvements in quality, safety and accessibility of healthcare. Nurses’ opinions regarding continuous monitoring appear to be positive. However, facilitators and barriers were little studied. This study explored nurses’ post-implementation experiences of the facilitators and barriers to continuously monitoring patients’ vital signs using a wireless device on general hospital wards. Methods: This study employed a cross-sectional survey. Vocational and registered nurses from three general wards in a Dutch tertiary university hospital participated in a survey comprising open and closed questions. The data were analysed using thematic analysis and descriptive statistics. Results: Fifty-eight nurses (51.3%) completed the survey. Barriers and facilitators were identified under four key themes: (1) timely signalling and early action, (2) time savings and time consumption, (3) patient comfort and satisfaction and (4) preconditions. Conclusions: According to nurses, early detection and intervention for deteriorating patients facilitate the use and acceptance of continuously monitoring vital signs. Barriers primarily concern difficulties connecting patients correctly to the devices and system.

## 1. Introduction

‘As the use of technological equipment becomes an ever-increasing component of nurses’ work, an adequate understanding of nurses’ attitudes towards medical devices in healthcare practice is an important precursor to the delivery of safe and effective care’ [1].

Wearable wireless devices, a device worn by patients and connected wirelessly to a monitor and the electronic patient record, were recently introduced in general hospital wards with the aim to improve patient outcomes and optimise nurses’ workflows [2,3]. Patients in an intensive care unit are already continuously monitored, opposite to patients admitted to a general ward and being monitored intermittently. Continuously monitoring vital signs of patients on general wards enhances patient safety compared to intermittent monitoring, by enabling nurses to recognise deteriorating patients earlier and provide a timely intervention [4,5]. A systematic review and meta-analysis confirmed these findings. However, it did not detect a significant effect on in-hospital mortality, unplanned intensive care unit admissions or the length of hospital stays [2]. A recent before-and-after study also demonstrated that continuously monitoring vital signs does not affect hospital stays and in-patient deaths. However, in contrast to the systematic review, a reduction in unplanned intensive care unit admissions and rapid response team calls was observed [6].

Keeping patients physically safe is one of the core elements of fundamental nursing care [7]. To achieve patient safety, nurses must monitor vital signs to measure, assess and interpret patients’ physiological well-being and act when signs of clinical deterioration appear [8]. Nurses’ experience and intuition are essential in making clinical judgements and recognising clinical deterioration early. Therefore, nurses’ visual and physical assessment of patients cannot be replaced by continuously monitoring vital signs alone [3,9,10,11]. Hence, continuously monitoring vital signs could complement nurses’ clinical judgement and empower decision-making and communication with doctors to enhance patient safety [12].

Successfully implementing healthcare technology depends on its adoption by individuals (i.e., that a user begins to use fully and routinely a new introduced technology), an organisation’s implementation strategy (i.e., process and factors that influence the adoption, spread and sustainability of the new technology) and their interdependence [13]. In addition, a healthcare technology’s use, acceptance, efficacy and quality and safety improvements are affected by nurses’ engagement and satisfaction and the technology’s perceived need and usefulness [13,14,15,16,17]. Insufficient engagement in health care technology (e.g., continuously monitoring vital signs), a lack of perceived ease of use and a lack of perceived usefulness of the device as described in the technology acceptance model (TAM), could affect the behavioural intention of nurses to use the new technology and could cause nurses to revert to previous behaviours, develop workarounds or avoid using the new technology [13,17,18]. This could negatively affect the implementation of the technology.

According to previous studies, nurses’ opinions of and engagement with continuously monitoring vital signs appeared to be positive. Stellpflug et al. [19] demonstrated that, according to nursing staff, continuous monitoring helps detect changes and deterioration in vital signs earlier, allowing them to treat deviating parameters promptly with appropriate interventions, stabilise patients and avoid the need for a rapid response team. Continuous monitoring also facilitates tracking ‘the effectiveness of interventions more efficiently’ [19].

A randomized controlled trial of our research group, conducted as a pilot study, demonstrated that, according to nurses, early detection of clinical deterioration and fewer actions required to measure vital signs facilitate the use of wireless technology to continuously monitor vital signs [11]. Additional positive experiences included enhanced decision-making [19], improved communication with doctors and other healthcare professionals [3,19], patient engagement in their care [3] and time savings, particularly during night shifts [3,20]. However, in addition to these positive results, mentioned barriers included the device’s size, design and functionality, alert fatigue caused by a multitude of (false) alarms, an assumed lack of personnel to monitor the data, nurses’ lack of expertise when interpreting continuous data and the potential for inappropriate escalations and a decline in patient attention [3,11].

Notably, these results were based on preliminary studies of limited duration. Moreover, a limited number of ward patients were continuously monitored during the study period. Therefore, the results were based on nurses’ initial experiences using wireless technology to monitor vital signs continuously and were primarily assumptions. Consequently, the results are uncertain, and further research is advised to guide future implementation strategies in general wards [1,3,11,19,20,21,22,23,24,25,26,27,28].

Currently, the continuous wireless monitoring of vital signs is common practice at several hospitals. Although wireless continuous monitoring of vital signs has been in use for some time, it is still relatively new and, therefore, is expected to have areas for improvement. Hence, understanding the actual factors that facilitate or impede its perceived usefulness and uptake in a general ward after working with continuous wireless monitoring for at least six months could support, extend or refute previous knowledge. This study explores the facilitators and barriers for nurses using a wireless device to monitor vital signs continuously in a general hospital ward in a post-implementation period. The results will help to smoothen the adoption and implementation of wireless devices for continuously monitoring vital signs and other promising technologies, to support and maintain high-quality care and committed and engaged nurses, now and in the future.

## 2. Materials and Methods

A cross-sectional survey comprising open and closed questions was performed to investigate nurses’ experiences using wireless technology to continuously monitor patients’ vital signs at a tertiary university hospital in the Netherlands. Continuous monitoring was implemented in three general wards: (1) abdominal and oncological surgery, (2) internal medicine and rheumatology and (3) gastroenterology. Continuous monitoring was introduced for six to fourteen months in all three wards and the scope of this study was to gain insight in nurses’ experiences; therefore, patients were not included. The ViSi Mobile Monitoring System was designed to monitor patients’ vital signs continuously in general care settings [29]. In addition to the fact that the ViSi Mobile design was intended for general wards, it was expected that patients in general care settings may benefit from continuous monitoring of their vital signs compared to intermittent monitoring of vital signs [2,5]. That is why this study was performed on three general wards.

All the participants in this study provided informed consent. Ethical approval was not required for this type of research according to Dutch law. The study was carried out in accordance with the Declaration of Helsinki [30] and the Netherlands’ General Data Protection Regulation. Three researchers (R.P.H.V., F.L.B.-V. and H.v.G.) had access to the anonymised survey results. In absence of a clinical trial, there was no need for registration in a clinical trial register. This study used the checklist for reporting of survey studies [31] (Appendix A).

### 2.1. Participants

Purposive sampling was employed. Included participants were vocationally educated nurses and registered nurses (n = 111) working in one of the three general wards and with regular practical experience using continuous monitoring devices. Therefore, senior nurses, nurse aids, temporary workers and interns were excluded.

### 2.2. The Device and Implementation

The ViSi Mobile Monitoring System was used to monitor patients’ vital signs continuously. The ViSi Mobile system is a patient-worn, battery-operated, portable device with a three-lead-wire electrocardiogram (ECG). It received a Conformité Européenne mark (CE) and Food and Drug Administration (FDA) approval for continuously monitoring the electrocardiogram, heart rate, blood oxygen saturation, non-invasive (continuous) blood pressure (cuff-based and cuff-less on a beat-to-beat-base) and skin temperature in hospital facilities [29]. The accuracy of novel devices is often still unclear because most devices still remain in the clinical validation and feasibility testing phase [32]. However, (pilot) studies showed that measurements of vital signs with wireless devices seemed to be consistent and correlate with nurses’ measurements of the patients’ respiratory rate, blood oxygen saturation, heart rate and/or blood pressure [33,34,35]. ViSi Mobile is able to measure blood pressure non-invasively and continuously on a beat-to-beat base which is new for general wards [29]. The cuff-based pressure measurements of the ViSi Mobile were validated against blood pressure measurements with Dinamap and results showed equivalence between both blood pressure measurements [36].

Vital signs were displayed on a wrist monitor and remote monitors placed at nurses’ stations. Vital signs were imported into the patient’s electronic health record (EHR) every minute. Transmitting vital signs from patients to remote viewers and the EHR was only possible with a wireless internet connection. A visible and audible alarm from the remote monitor indicates when vital signs deviate.

The implementation of continuous wireless monitoring with ViSi Mobile started after a pilot study in which the ViSi Mobile was pilot tested between December 2014 and March 2015 [33] and before the start of studying the effect of continuous wireless vital sign monitoring on unplanned ICU admissions and rapid response team calls in August 2018 [6]. With the start of the intervention period in the above mentioned study of Eddahchouri, Peelen, Koeneman, Touw, van Goor and Bredie [6], the ViSi Mobile was implemented in May 2018 in the abdominal and oncological surgery ward and the internal medicine and rheumatology ward. All nurses received a mandatory face-to-face training by a product specialist of Sotera (a former nurse) which lasted for two hours. This product specialist spent two weeks in the hospital to support nurses on both wards working with ViSi Mobile. Twelve nurses, six nurses of each ward working in direct patient care or functioning as senior nurse, volunteered to be a superuser of the ViSi Mobile. These superusers received in-depth information by this product specialist about the technical details of the device with the aim that superusers could inform, supervise and give bed-side training to nurse colleagues. Thereafter, superusers organised refresher training courses twice at the request of the nurses and during all shifts superusers remained points of contact around working with ViSi Mobile.

In January 2019, ViSi Mobile was implemented in the gastroenterology ward. Six superusers of the abdominal and oncological surgery ward guided this implementation and they organized a half day training session for all nurses of the gastroenterology ward. In the gastroenterology ward, two nurses volunteered to be a superuser and these superusers were informed and trained by the superusers of the abdominal and oncological surgery ward. These superusers were the point of contact for their own colleagues. Between May 2018 and January 2019, the superusers developed a poster with common information about how to connect patients to the ViSi Mobile and daily appointments (e.g., when to change batteries). At the same time, Sotera developed an e-learning module that taught users how to work with the ViSi Mobile. All nurses of the three wards were obliged to complete this e-learning. In the meantime, nurses working with ViSi Mobile received diverse tasks and responsibilities (see Appendix B for a detailed description).

Continuous monitoring of vital signs was possible for all patients in the three wards (18 abdominal and oncological surgery beds, 25 internal medicine and rheumatology beds and 13 gastroenterology beds). Several patients were not connected to ViSi Mobile due to underlying conditions (see Appendix B).

### 2.3. Data Collection

A ten-item survey was conducted to obtain an initial impression of the facilitators and barriers to wireless continuous monitoring and using ViSi Mobile experienced by nurses in daily practice. Although, survey research has limitations such as non-response or missing answers, there are also specific reasons to use a survey [37]. In this study, a survey was chosen to give all nurses the opportunity to share their experiences of continuous monitoring of vital signs with the ViSi Mobile, so that a broad picture of the facilitating and impeding factors could be formed in a short period. The questions were formulated by seven experts from different backgrounds: two innovation experts, two nurse researchers working with continuous monitoring and three physicians. The questions were derived from individual experiences of the nurse researchers and physicians working with continuous monitoring and ViSi Mobile and from information provided during informal discussions with nurses from the three wards. The survey was submitted to one nurse for testing and refinements, after which minor adjustments were made. All the experts agreed on the final version of the questionnaire.

Seven of the ten survey items included a primary closed question and a secondary open question to clarify or complement the primary response. The remaining three items comprised only open questions, resulting in ten open questions in total. The survey is presented in Appendix C. All the participants were invited by e-mail to complete the digital survey using SurveyMonkey. Survey completion was possible from the first invitation on 18 July 2019 until 23 August 2019. Reminders were sent to all the participants two weeks after the first invitation. Each respondent could complete the questionnaire a maximum of once to prevent multiple participation, regulated by SurveyMonkey’s settings.

### 2.4. Data Analysis

This survey employed mixed-methods data analysis. Descriptive statistics were used to describe the respondents’ and non-respondents’ demographics and quantitatively analyse the responses to the closed questions. Qualitative thematic analysis was used to evaluate the responses to the open questions. The non-respondents’ demographics were based on the known characteristics of the invited group minus the responding group’s characteristics.

Thematic analysis was selected to identify, analyse and report themes within the data generated by the ten open questions [38,39]. This analysis highlighted the most common themes, providing an answer to the research question without quantification [39]. The answers to the open questions were extracted from SurveyMonkey to the computer-assisted qualitative data analysis software program ATLAS.ti (ATLAS.ti Scientific Software Development GmbH, Berlin, Germany, version 8.4.20). Two researchers (R.P.H.V. and F.L.B.-V.) performed the thematic analysis independently. Following the individual analyses, differences in the findings were discussed until a consensus was reached regarding the emerging themes. The themes were discussed and jointly approved by all the study authors. Researcher triangulation increased the study’s reliability and credibility [40,41]. Corrections made during the consensus process were reported to increase transparency and confirmability [40].

### 2.5. Data Presentation

Following thematic analysis, the themes were described independently and determined the structure in the results section. Where applicable, the quantitative outcomes of the closed questions related to the theme were added to the description to clarify the findings regarding facilitators and barriers.

## 3. Results

Within the three departments, 111 nurses were invited to participate in the survey. Fifty-eight nurses (51.3%) completed the survey. Fifty-three (48.7%) nurses did not respond or declined to participate (no reason was given). The respondents’ and non-respondents’ demographics are presented in Table 1.

The response rate for the primarily closed questions was 100%. The open questions had an average response rate of 75%.

Four themes emerged from the thematic analysis of the open questions: (1) timely signalling and early action, (2) time savings and time consumption, (3) patient comfort and satisfaction and (4) preconditions. The preconditions theme comprises five subthemes: (1) device design and technical concerns, (2) vital signs’ reliability, (3) internet connectivity, (4) nurses’ knowledge and training and (5) logistics.

### 3.1. Theme 1: Timely Signalling and Early Action

**Figure 1:**
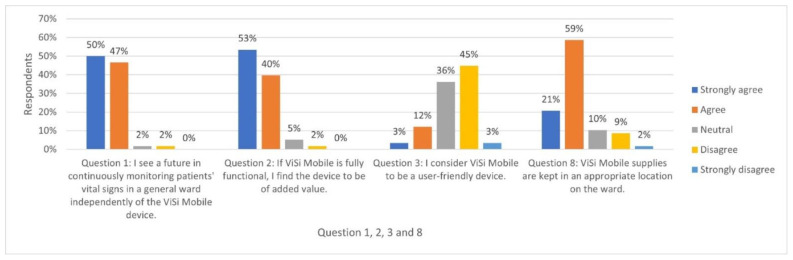
Nurses’ experiences of continuous monitoring, the ViSi Mobile system and its logistics.

The first theme concerns timely signalling and early intervention when patients’ vital signs deviate. Most nurses experienced direct and continuous insight into their patients’ vital signs, enabling them to detect trends and compare and interpret current and previous measurements. The respondents could recognise deterioration in patients’ vital signs at an early stage and prevent or alleviate adverse events through timely recognition and intervention. For example, Respondent 4 commented, ‘Last week, we had a successful resuscitation because ViSi Mobile showed a low heart rate’. Due to these experiences, 97% of the nurses were positive about continuously monitoring vital signs, and 93% of the nurses considered ViSi Mobile to provide added value when the device functioned adequately (Figure 1). Most nurses mentioned early recognition and intervention in deteriorating patients as the most critical advantages of continuous monitoring using ViSi Mobile. Respondent 10 commented, ‘This allows you to respond/act appropriately and in a timely manner when the device shows abnormal values and is not acted upon until the next monitoring moment’.

### 3.2. Theme 2: Time Savings and Time Consumption

Nurses found continuous monitoring time-saving and time-consuming. Most nurses mentioned that continuous monitoring accelerated their recognition of deteriorating vital signs and early intervention. However, they did not mention how much time could be saved as a result.

Nurses mentioned that continuous monitoring saved time because it was easier and quicker to load vital signs into the EHR using ViSi Mobile than input them manually. Nurses considered this feature and the potential to detect deteriorating patients early the most significant reasons ViSi Mobile added value in nursing practice (Figure 1).

**Figure 2:**
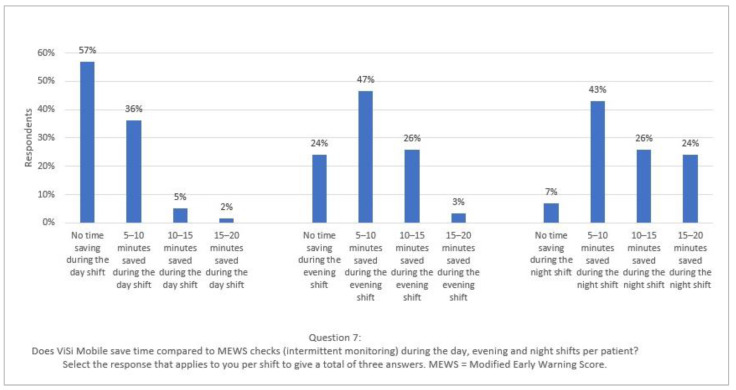
An overview of the time saved by continuous monitoring per shift, as experienced by nurses.

Time savings were most notable in the evening and night shifts (mentioned by 76% and 93% of the respondents, respectively). According to 57% of the nurses, ViSi Mobile did not save time during the day (see Figure 2). The difference in time savings between shifts was due to higher patient/nurse ratios during the evening and night shifts. Therefore, quickly loading more patients’ vital signs into the EHR resulted in greater absolute time savings.

Nurses gave multiple reasons for the time-consuming element of continuous monitoring during day shifts: assigning and connecting patients to the devices, calibrating devices, replacing parts and troubleshooting. These actions are necessary to ensure that devices function adequately and all patients on the ward are connected. They were particularly time-consuming during the day shift because patients showered in the morning and must be reconnected afterwards. In addition, stickers must be replaced twice weekly (by appointment during the day shift) or when necessary (e.g., after heavy sweating during the night). Moreover, troubleshooting must be transferred from the night to the day shift to avoid waking patients (e.g., to restore a dysfunctional device). Furthermore, new patients were primarily admitted to the ward during the day. Respondent 43 commented, ‘On the day shift, you are often also connecting new patients, reinstalling ViSi Mobiles that no longer work, which was not done in the evening/night’.

Several respondents implied that continuous monitoring using ViSi Mobile saved time only when the device functioned adequately. Otherwise, it was more time-consuming. As demonstrated in Figure 1, 48% of the nurses did not consider the device user-friendly because it did not always function fully due to faulty materials or poor internet connection:

*In the day and evening shift, it does give you some gain, but usually only 5–10 min because it almost never happens that all three to six are functioning. In the evening shift, maybe sometimes a little more time gain, because you have more patients there and especially when they all function. On the night shift, the morning round, it certainly saves a lot of time. But also only when they are functioning; otherwise, you still have to calibrate them or manually measure the controls.* (Respondent 18)

**Figure A1:**
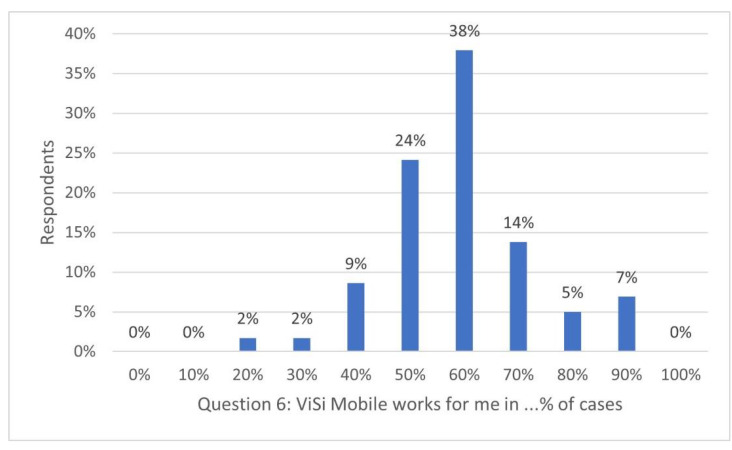
Overview of how often ViSi Mobile works based on nurses’ experiences.

Consequently, time was spent troubleshooting and repairing the system. Figure A1 (Appendix D) demonstrates how often ViSi Mobile worked adequately according to nurses.

### 3.3. Theme 3: Patient Comfort and Satisfaction

The third theme explores nurses’ perceptions of patient comfort and satisfaction with continuous monitoring. Positive and negative perceptions emerged. Some nurses observed improved patient comfort and satisfaction using ViSi Mobile. Nurses assumed two benefits for their patients: fewer measurements, especially at night, and less inconvenience when measuring blood pressure because it is not necessary to inflate a blood pressure cuff three times a day (or up to every hour according to the modified early warning score (MEWS) protocol followed by the hospital). Due to these benefits, nurses perceived that continuous wireless monitoring added value to patient care (Figure 1). Respondent 52 commented, ‘We don’t need to disturb patients as often, especially at night. Patients sleep better because of this, I think’.

Several nurses observed that ViSi Mobile disturbed patients’ comfort because its design can hinder them during daily activities and during their sleep. In particular, nurses considered the number of cables ‘too much’ and the wrist monitor ‘too big’ and ‘too heavy’. Respondent 24 stated, ‘I find it user-friendly, but patients find the battery on the arm too big, the stickers too tight on the skin, the cables inconvenient when washing, dressing and showering. And when sleeping, the chest unit bothers them’. Hence, nurses questioned ViSi Mobile’s user-friendliness from the patient’s perspective (Figure 1). Two nurses commented that patients could become restless or obsessed with seeing their vital signs. Respondent 48 commented, ‘Some patients get restless from the data they can see, the beeping of the device, let alone the many cords, adhesives and the fairly heavy device that was attached to the body’.

### 3.4. Theme 4: Preconditions

The fourth theme explores the absence of or desire for certain preconditions when working with continuous monitoring, as experienced by nurses. These preconditions were divided into five subthemes: (1) device design and technical concerns, (2) vital signs’ reliability, (3) internet connectivity, (4) nurses’ knowledge and training and (5) logistics.

#### 3.4.1. Device Design and Technical Concerns

Nurses observed that the device did not function appropriately all the time (Appendix D, Figure A1) and assumed that this was due to the device’s design. Nurses largely attributed the device’s poor performance to malfunctioning thumb or chest sensors due to broken cables. In addition, several nurses encountered the following inconveniences when using the device: low battery power, problem-solving difficulties, chest cables and ECG stickers that inadvertently detach too quickly, problems calibrating the device and patient-related factors (e.g., atrial fibrillation, scleroderma and the patient’s position in bed).

The most desired improvement was to optimise ViSi Mobile’s design and functioning. The respondents would like to see the following improvements: softer, smaller and lighter materials and equipment (e.g., batteries), fewer cables, more robust cables in the thumb and chest sensors, less sensitivity to daylight in the thumb sensor and improved adherence and covering patches to shield the thumb sensor from daylight, better ECG-sticker adhesion, more hygienic thumb patches and longer battery life.

#### 3.4.2. Vital Signs’ Reliability

Nurses occasionally doubted the measured vital signs’ reliability. Doctors experienced similar doubts due to differences in measurements with older devices used to measure vital signs. In particular, nurses and doctors questioned the reliability of blood pressure and oxygen saturation measurements. Doubts also arose due to problems connecting patients to ViSi Mobile and calibrating the device. Several nurses commented that doubts about the reliability of vital sign measurements taken by ViSi Mobile resulted in doctors’ orders or nurse initiatives to measure vital signs using older equipment, such as device for indirect non-invasive automated MAP measurement, and to rely on those measurements. Respondent 48 commented: ‘… also the saturation often deviates or is not present. Why should I rely on the rest?’

#### 3.4.3. Internet Connectivity

Most of the respondents commented that improved internet connectivity is a requirement. Nurses noted disruptions in wireless internet connectivity on wards and specific patient rooms over time. According to nurses, disruption in internet connectivity reduced battery life and caused problems calibrating devices and assigning patients when changing batteries. Therefore, respondents would like to calibrate and assign patients more easily and quickly by optimising internet connectivity.

#### 3.4.4. Nurses’ Knowledge and Training

**Figure A2:**
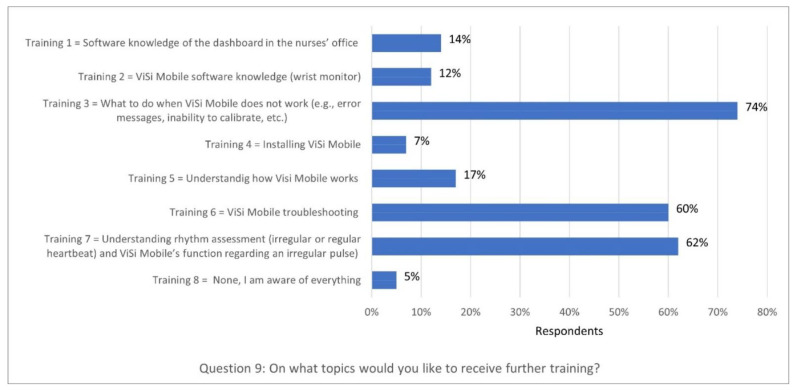
What nurses want to learn about continuous monitoring using ViSi Mobile.

Several nurses mentioned training as one of the top three improvements that should be prioritised when working with ViSi Mobile. Three topics emerged in training nurses to use wireless continuous monitoring (Appendix D, Figure A2). Nurses wanted to know what to do when ViSi Mobile did not work and how to identify malfunctions. They also wanted a brief explanation of rhythm assessment (irregular or regular heartbeat) and ViSi Mobile’s function in the case of an irregular pulse. Respondent 6 commented, ‘To my understanding, many patients aren’t connected because we, ourselves as nurses, can’t resolve the error messages’.

Respondents were divided about how they would like to receive training. Most respondents preferred a presentation, and others preferred e-learning or a combination of the two. Several nurses desired the ability to re-read instructions or training materials after training and the ability to ask questions during presentations. A minority wanted a paper reference manual.

#### 3.4.5. Logistics

Most respondents felt that ViSi Mobile supplies were stored in a suitable location on the ward (Figure 1). Common wishes were centralising materials storage, a mobile container with the necessary equipment to take into patients’ rooms and arrangements to optimise material and stock management and corresponding responsibilities. Respondents also desired sufficient functioning equipment and materials to replace broken parts.

## 4. Discussion

The survey results demonstrated that most nurses were positive towards using wireless technology to monitor vital signs continuously in a general ward. The factors facilitating continuous wireless monitoring emerged under two themes: (1) timely signalling and early action and (2) time savings and time consumption. Nurses experienced direct and continuous insight into patients’ vital signs, the potential to prevent and alleviate adverse events through timely recognition and intervention and time savings during the evening and night shifts.

The main barrier was encountered under the preconditions theme, notably under two subthemes: (1) device design and technical concerns and (2) internet connectivity. Nurses experienced problems with the availability of materials and ensuring that devices were working effectively due to fragile parts, limited stock, poor internet connectivity and a lack of knowledge to solve problems. Nurses experienced facilitators (e.g., fewer measurements and less patient disturbance) and barriers (e.g., heavy devices) under the patient comfort and satisfaction theme. Despite the mentioned barriers, including perceived ease of use which is specifically found in theme ‘Preconditions’, the perceived usefulness found in theme ‘Timely signalling and early action’ makes nurses willing to continue using continuous monitoring of vital signs with a wireless device. That this perceived usefulness outweighs perceived ease of use, and positively influences attitude, acceptance and behavioural intention of nurses to use the new technology, is in line with the technology acceptance model [17].

The findings of this study were similar to the results of a pilot study by Weenk, Bredie, Koeneman, Hesselink, van Goor and van de Belt [11] and a systematic review of the adoption of information and communication technologies (ICT) by healthcare professionals [42]. The systematic review identified the system’s perceived usefulness, ease of use and time efficiency as factors facilitating ICT adoption and implementation. Barriers to adopting and implementing ICT included time constraints, workload, reservations about the quality, completeness and relevance of resources and design and technical concerns [42].

This study revealed several additional findings. Firstly, although nurses expected efficient health care following the introduction of continuous vital sign monitoring, nurses did not specify when and how efficiency would be achieved [11]. This study revealed that nurses primarily experienced time savings during the evening and night shifts. Time saved during the evening and night shifts could facilitate the adoption and implementation of health care technology, such as continuously monitoring vital signs [13,42,43]. However, it was unclear how time savings were achieved and how it affected nursing care since nurses only mentioned that loading vital signs into the EHR was easier and quicker than manually inputting them. The pilot study assumed that the time saved would be invested in patients. However, the post-implementation results did not link saved time with time for patients. Additionally, it should be noted that it was difficult to objectify the nursing time required to monitor vital signs and make comparisons between wards or hospitals, due to different methods of measuring vital signs, calculating early warning scores and recording and timing patient record entries [27].

Secondly, as expected in several studies [3,9,10,11,12,44,45], nurses did not mention any adverse impact on nurse–patient relationships or fewer interactions with patients following the introduction of continuous wireless monitoring. A possible explanation is that nurses visit patients to connect them to or calibrate ViSi Mobile and observe their patients when measuring vital signs.

Thirdly, half of the respondents mentioned the positive influence of continuous monitoring on healthcare quality and safety during the pilot study [11]. Conversely, almost all the nurses mentioned it during the post-implementation phase. In both studies, nurses mentioned that the potential for early detection and intervention when vital signs deteriorated enhanced healthcare quality and safety. This finding indicates that more nurses experienced the positive effects of continuous monitoring on healthcare quality and safety when using ViSi Mobile for an extended period. The potential to improve quality and safety of health care seemed to heighten nurses’ motivation to use continuous monitoring. Although, nurses’ motivation to use continuous monitoring depended on prerequisites as well (e.g., the integration and compatibility with clinical workflow, available training and alarm management) [28].

Interestingly, nurses did not mention that technology, such as continuous monitoring, could play an important role in the near future to face the challenge of the growing demand for healthcare and the nursing shortages. New technologies are thought to be crucial for continued patient care and the healthcare system at large [46]. The prospect of the future, in which certain technologies can make patient care easier and more efficient and help nurses manage their large patient loads, needs to be brought more to the attention of nurses.

Understanding the facilitators and barriers to adopting wireless technology for continuously monitoring vital signs will help optimise its implementation in other wards and hospitals [13,23]. Therefore, three recommendations are made.

First, attention must focus on the technological innovation’s characteristics. In addition, end-users (i.e., nurses and patients) must be involved in the process at the outset by designing and developing the device (co-creation) and implementing and evaluating the technology [47]. Allowing stakeholders to make informed decisions and reach consensus helps implement wireless continuous monitoring that is appropriate for the complex demands of healthcare practice and end-user preferences [23,42,47,48].

The second recommendation concerns prerequisites. Successful implementation depends on sufficient available and competent professionals to guide the process. Furthermore, risk analysis must be conducted to create and share a vision of continuous monitoring within the organisation and clarify expectations and responsibilities regarding device storage and management, troubleshooting and communication with manufacturers. Arrangements must be made to ensure equipment maintenance and that devices are working correctly [49].

The third recommendation is to provide adequate support and training, including supervisors, to ensure that all nurses have the skills to solve the most common problems concerning the technology [3,15,42,48,50]. Tailored training, learning on the job and peer support can assist nurses lacking digital knowledge [51]. Furthermore, training must be available for all nurses before, during and after implementation.

Future research should focus on the implications of continuously monitoring vital signs for nursing practice. It would be interesting to understand how continuously monitoring vital signs influences nurses’ clinical judgement and decision-making [12]. Further knowledge about the reliability, validity and feasibility of continuous monitoring, including clinical and cost outcomes and the technical problems associated with the monitoring system’s connectivity, could inform health care professionals and guide implementation on a larger scale (e.g., in hospital and home-care settings) [32,52].

The feasibility of continuously monitoring vital signs using ViSi Mobile in a home-care setting is a particularly valuable research topic. Different devices provide diverse opportunities for continuous monitoring in hospital and home-care settings [32]. Moreover, nurses prefer to have a complete set of parameters when monitoring patients inside and outside the hospital [50]. Since ViSi Mobile provides a complete set of parameters, including non-invasive blood pressure measurement, this device has the potential for implementation in a home-care setting.

Future research should also focus on if and how continuous monitoring on general wards influences nursing practice, because it was unclear whether time savings meant more time for patients. Whether saved time can affect nursing-sensitive outcomes in patients and the degree of “care left undone” is also an interesting question.

Finally, research should consider how to design a more patient-friendly, comfortable and robust device that is cheaper, more sustainable and less work intensive for nurses without compromising patient safety. Addressing these questions could make a difference in nurses’ daily working processes and positively influence patient outcomes.

### Limitations

A study limitation was the results’ limited generalizability due to the single-centre design, relatively few nursing participants and the use of one single device. In addition, selection bias cannot be excluded because higher educated nurses were more willing to respond to the survey (62% registered nurses vs. 32% vocational nurses) and the average age of the respondents was about seven years below the average age of the non-respondents (29.6 vs. 36.6 years old). Moreover, respondents differed in their experience working with the device, from six months (gastroenterology) to fourteen months (abdominal and oncological surgery and internal medicine and rheumatology). Furthermore, the data were collected using one questionnaire, and expanding upon the closed questions with a comment was optional. Therefore, relevant experiences could have been missed, and data saturation was not reached. It is unclear whether this could have influenced the results. The last limitation that could be mentioned is the absence of a framework in the study design. In this study, we chose not to use a framework because an inductive way of coding was well applicable to answer the practically oriented question of this study [53]. To assess and compare results from different studies that identify the factors contributing to the adoption of a new technology, a framework was advised in the research design of a study [54]. In addition to the existing technology acceptance model (TAM) and the unified theory of acceptance and use of technology (UTAUT), a revised UTAUT was produced, the so called ‘meta-analysis-based-modified version of UTAUT (meta-UTAUT)’ [55]. This revision of the UTAUT framework showed that an individual’s attitude has a partially mediating effect on “performance expectancy, effort expectancy, facilitating conditions, and social influence on behavioural intention, and also has a direct effect on usage behaviour” [56]. Inclusion of ‘attitude’ in a framework could possibly clarify differences between individuals behavioural intention to use and actual behavioural use regarding continuous monitoring of vital signs [55]. Therefore, this meta-UTAUT framework might be useful in future studies.

## 5. Conclusions

To support and maintain high quality of care, it is important to know the facilitators and barriers which influence the adoption and implementation of new technologies. This study aimed to provide the facilitators and barriers for nurses using wireless devices to monitor vital signs continuously in a general hospital ward in a post-implementation period. This study confirmed that in the post-implementation phase, nurses on general wards support continuously monitoring vital signs and experience it as helpful in their daily working process. Continuously monitoring vital signs provides added value by maintaining patient safety and quality care, making it a significant facilitator for adopting and using the technology. Nurses do not mention a negative impact on nurse–patient relationships following the introduction of continuous monitoring. Continuously monitoring vital signs is associated with time savings during evening and night shifts. However, most nurses found it time-consuming during day shifts. The main reason was difficulties ensuring that all the devices were working effectively due to the device’s design and intermittent internet connectivity. However, it was unclear how time savings and consumption affect nursing practice. It is necessary to overcome the barriers discussed above and leverage positive experiences to increase the technology’s adoption and its advantages and efficiencies for patients and nurses.

This study distinguished itself from earlier studies because it covered a fourteen-month post-implementation period, in which continuous wireless monitoring using a specific device was carried out in daily nursing practice rather than as a trial [3,9,11,33,42,57].

### Relevance to Clinical Practice

According to nurses’ experiences, wireless technology for continuously monitoring vital signs is essential for delivering fundamental care; it maintains patient safety by recognising deteriorating patients early, enabling prompt intervention, compared to intermittently monitoring vital signs. In addition, nurses find continuous vital sign monitoring time-saving during evening and night shifts and occasionally during the day when problems are resolved.

This time-saving component could be part of a solution to deliver safe and efficient care to more patients with fewer caregivers due to an ageing population and nursing staff shortages [58]. The potential to monitor vital signs remotely in a home-care setting could result in earlier patient discharge from hospital [50] and, thus, be part of the solution to address the above challenge. Finally, to fully benefit from the advantages of continuous monitoring, support nurses and keep them engaged and support implementation at other sites, the barriers mentioned in this study must be addressed at an early stage.

## Figures and Tables

**Table 1:** Respondent and non-respondent demographics.

	Respondents n = 58 (%)	Non-Respondents n = 53 (%)
Gender		
Male (%)	9 (15.5)	8 (15.1)
Female (%)	49 (84.5)	45 (84.9)
Education		
Vocational educated nurse (%)	12 (20.7)	25 (47.2)
Registered nurse (%)	46 (79.3)	28 (52.8)
Ward		
Abdominal/oncological surgery (%)	22 (37.9)	18 (34.0)
Internal medicine/rheumatology (%)	27 (46.6)	25 (47.2)
Gastroenterology (%)	9 (15.5)	10 (18.9)
Age group		
21–30 years (%)	42 (72.4)	26 (49.1)
31–40 years (%)	10 (17.2)	9 (17.0)
41–50 years (%)	3 (5.2)	8 (15.1)
51–60 years (%)	3 (5.2)	7 (13.2)
61–64 years (%)	0 (0)	3 (5.7)

## Data Availability

The data presented in this study are available on request from the corresponding author. The data are not publicly available due to the way data were collected and stored on the local network of the medical centre as a result of common practices during this study.

## Appendix A. Checklist for Reporting of Survey Studies (CROSS) [31]

**Table:**

Section/Topic	Item	Item Description	Reported on Page #
**Title and abstract**			
Title and abstract	1a	State the word “survey” along with a commonly used term in title or abstract to introduce the study’s design.	#1
	1b	Provide an informative summary in the abstract, covering background, objectives, methods, findings/results, interpretation/discussion, and conclusions.	#1
**Introduction**			
Background	2	Provide a background about the rationale of study, what has been previously done, and why this survey is needed.	#1–3
Purpose/aim	3	Identify specific purposes, aims, goals, or objectives of the study.	#2–3
**Methods**			
Study design	4	Specify the study design in the methods section with a commonly used term (e.g., cross-sectional or longitudinal).	#3
Data collection methods	5a	Describe the questionnaire (e.g., number of sections, number of questions, number and names of instruments used).	#4–5 + Appendix C
	5b	Describe all questionnaire instruments that were used in the survey to measure particular concepts. Report target population, reported validity and reliability information, scoring/classification procedure, and reference links (if any).	N/A
	5c	Provide information on pretesting of the questionnaire, if performed (in the article or in an online supplement). Report the method of pretesting, number of times questionnaire was pre-tested, number and demographics of participants used for pretesting, and the level of similarity of demographics between pre-testing participants and sample population.	#4–5
	5d	Questionnaire if possible, should be fully provided (in the article, or as appendices or as an online supplement).	Appendix C
Sample characteristics	6a	Describe the study population (i.e., background, locations, eligibility criteria for participant inclusion in survey, exclusion criteria).	#3
	6b	Describe the sampling techniques used (e.g., single stage or multistage sampling, simple random sampling, stratified sampling, cluster sampling, convenience sampling). Specify the locations of sample participants whenever clustered sampling was applied.	#3
	6c	Provide information on sample size, along with details of sample size calculation.	#3–4 + N/A
	6d	Describe how representative the sample is of the study population (or target population if possible), particularly for population-based surveys.	N/A
Survey administration	7a	Provide information on modes of questionnaire administration, including the type and number of contacts, the location where the survey was conducted (e.g., outpatient room or by use of online tools, such as SurveyMonkey).	#4–5
	7b	Provide information of survey’s time frame, such as periods of recruitment, exposure, and follow-up days.	#4–5
	7c	Provide information on the entry process:	
		- For non-web-based surveys, provide approaches to minimize human error in data entry.	N/A
		- For web-based surveys, provide approaches to prevent “multiple participation” of participants.	#5
Study preparation	8	Describe any preparation process before conducting the survey (e.g., interviewers’ training process, advertising the survey).	#4–5
Ethical considerations	9a	Provide information on ethical approval for the survey if obtained, including informed consent, institutional review board [IRB] approval, Helsinki declaration, and good clinical practice [GCP] declaration (as appropriate).	#3
	9b	Provide information about survey anonymity and confidentiality and describe what mechanisms were used to protect unauthorized access.	#3
Statistical analysis	10a	Describe statistical methods and analytical approach. Report the statistical software that was used for data analysis.	#5
	10b	Report any modification of variables used in the analysis, along with reference (if available).	N/A
	10c	Report details about how missing data was handled. Include rate of missing items, missing data mechanism (i.e., missing completely at random [MCAR], missing at random [MAR] or missing not at random [MNAR]) and methods used to deal with missing data (e.g., multiple imputation).	#5
	10d	State how non-response error was addressed.	N/A
	10e	For longitudinal surveys, state how loss to follow-up was addressed.	N/A
	10f	Indicate whether any methods such as weighting of items or propensity scores have been used to adjust for non-representativeness of the sample.	N/A
	10g	Describe any sensitivity analysis conducted.	N/A
**Results**			
Respondent characteristics	11a	Report numbers of individuals at each stage of the study. Consider using a flow diagram, if possible.	#5 (Table 1)
	11b	Provide reasons for non-participation at each stage, if possible.	N/A
	11c	Report response rate, present the definition of response rate or the formula used to calculate response rate.	#5
	11d	Provide information to define how unique visitors are determined. Report number of unique visitors along with relevant proportions (e.g., view proportion, participation proportion, completion proportion).	N/A
Descriptive results	12	Provide characteristics of study participants, as well as information on potential confounders and assessed outcomes.	#5–6
Main findings	13a	Give unadjusted estimates and, if applicable, confounder-adjusted estimates along with 95% confidence intervals and *p*-values.	N/A
	13b	For multivariable analysis, provide information on the model building process, model fit statistics, and model assumptions (as appropriate).	N/A
	13c	Provide details about any sensitivity analysis performed. If there are considerable amount of missing data, report sensitivity analyses comparing the results of complete cases with that of the imputed dataset (if possible).	N/A
**Discussion**			
Limitations	14	Discuss the limitations of the study, considering sources of potential biases and imprecisions, such as non-representativeness of sample, study design, important uncontrolled confounders.	#12
Interpretations	15	Give a cautious overall interpretation of results, based on potential biases and imprecisions and suggest areas for future research.	#10–12
Generalizability	16	Discuss the external validity of the results.	#12
**Other sections**			
Role of funding source	17	State whether any funding organization has had any roles in the survey’s design, implementation, and analysis.	#13
Conflict of interest	18	Declare any potential conflict of interest.	#13
Acknowledgements	19	Provide names of organizations/persons that are acknowledged along with their contribution to the research.	#13

## Appendix B. Working with ViSi Mobile

ViSi Mobile, with a three-lead-wire electrocardiogram, provides continuous wireless monitoring at a tertiary university hospital in the Netherlands. The ViSi Mobile system received a CE mark and FDA approval for continuously monitoring the electrocardiogram, heart rate, blood oxygen saturation, non-invasive blood pressure (cuff-based and cuff-less on a beat-to-beat-base) and skin temperature in hospital facilities (Sotera Wireless, n.d.). Three leads and a wrist monitor are connected to the patient.

Vital signs are displayed on the wrist monitor and remote monitors at nurses’ stations. Vital signs are imported into the patient’s electronic health record (EHR) every minute. A healthcare professional stores vital signs in the EHR, imported vital signs that are not stored, are deleted after discharge.

Transmitting vital signs from patients to the remote viewers and EHRs is only possible with a wireless internet connection. Therefore, a patient can go anywhere in the hospital while remote monitoring continues. When wireless internet connectivity is lost, vital signs remain visible on the wrist monitor.

The wards included in this study use the Modified Early Warning Score (MEWS) to interpret patients’ physiological states to detect potential or established critical illness [59]. Current hospital-wide and ward-specific protocols dictate that nurses measure vital signs three times daily and act on them according to MEWS guidelines. ViSi Mobile primarily generates an early warning score automatically. A core temperature measurement and manual check of the pulse rate’s regularity are necessary to complete the score. The remote viewer generates a visible and audible alarm when vital signs deviate. The system has default alarm limits that clinicians can adjust manually to provide individualized care [60]. When vital signs deviate, a nurse checks the vital signs trend and calls the first nurse responsible for the patient, who is assessed. Alarms only appear on the remote viewers and must be observed by a nurse.

All adult patients in the three wards are connected to ViSi Mobile. Exclusion from continuous monitoring must be considered when patients are focused on their vital signs, need to be cared for without stimuli, have skin problems or are receiving palliative care.

Nurses working with ViSi Mobile have several tasks and responsibilities: assigning and connecting patients to the ViSi Mobile system, changing batteries twice daily, checking that ViSi Mobile is working correctly during their shift, changing stickers twice a week and calibrating the device every 24 hours. Besides daily calibration, recalibration is sometimes necessary, for example, after administering vasoactive drugs or when the device instructs recalibration due to changes in blood pressure or ECG patterns [60]. Dedicated nurses usually help solve more complex errors.

## Appendix C. The Survey

A ten-item survey was conducted to collect information about the facilitators and barriers to wireless continuous monitoring and using ViSi Mobile in daily nursing practice. Seven items comprised a primary closed question and a secondary open question to clarify or complement the primary response; three items comprised open questions, resulting in ten open questions in total. All the subjects were invited to complete the digital survey using SurveyMonkey between the first invitation on 18 July 2019 and 23 August 2019.

The respondents were asked to respond to the following statements and questions:

Question 1:

- I see a future in continuously monitoring patients’ vital signs in a general ward independently of the ViSi Mobile device *.
- Explain why you do or do not see a future in continuously monitoring patients’ vital signs.

Question 2:

- If ViSi Mobile is fully functional, I find the device to be of added value *.
- Explain this answer.

Question 3:

- I consider ViSi Mobile to be a user-friendly device *.
- If you find ViSi Mobile a less user-friendly device, explain why.

Question 4:

- If ViSi Mobile is fully functional, the main benefits are (give a maximum of three benefits).

Question 5:

- If ViSi Mobile does not function properly, it is often due to (give a maximum of three answers).

Question 6:

- ViSi Mobile functions for me in …% of cases (please give an estimation between 0% and 100%).
- Give an explanation, if possible.

Question 7:

- Does ViSi Mobile save time compared to MEWS checks (intermittent monitoring) during the day, evening and night shifts per patient? Select the response that applies to you per shift to give a total of three answers. MEWS = Modified Early Warning Score.

*Response categories (answer per shift):*

- No time saving during the day/evening/night shift
- 5–10 min saved during the day/evening/night shift
- 10–15 min saved during the day/evening/night shift
- 15–20 min saved during the day/evening/night shift

- If the answer is no, indicate how much more time you spend on average per patient, per shift, compared to your previous situation.

Question 8:

- ViSi Mobile supplies are kept in an appropriate location on the ward *.
- Indicate what you would like to see change on the ward concerning stock and location.

Question 9:

- On what topics would you like to receive further training?

*Response categories:*

- Software knowledge of the dashboard in the nurses’ office
- ViSi Mobile software knowledge (wrist monitor)
- What to do when ViSi Mobile does not work (e.g., error messages, inability to calibrate, etc.)
- Installing ViSi Mobile
- Understanding how ViSi Mobile works
- ViSi Mobile troubleshooting
- Understanding rhythm assessment (irregular or regular heartbeat) and ViSi Mobile’s function regarding an irregular pulse
- None, I am aware of everything

- How would you like to receive this continuing education?

Question 10:

- What do you consider the main areas of improvement that should be prioritised regarding ViSi Mobile (give a maximum of three answers)?

* Response options: completely disagree, disagree, neutral, agree and completely agree.

## Appendix D. Figures for Closed Questions 6 and 9

A ten-item survey collected information about facilitators and barriers to wireless continuous monitoring and using ViSi Mobile in daily nursing practice (Appendix C). The results for closed questions 6 and 9 are given in Figure A1 and Figure A2.

## Appendix E. Relevance to Clinical Practice

Nurses are positive towards continuously monitoring vital signs as it enhances healthcare quality and safety, saves time in evening and night shifts and has the potential to support nursing care in hospital and home-care settings. Continuous monitoring offers the possibility to maintain the accessibility of healthcare in times of aging population and nursing shortages. This study highlights the barriers that must be overcome and makes recommendations for optimising future implementation of promising technologies to support and maintain high-quality care and committed and engaged nurses.

## Appendix F. Impact Statement

What does this paper contribute to the wider global clinical community?

- Nurses are positive towards using remote wireless technology to monitor vital signs continuously because it enables them to detect and react to clinical deterioration in patients early. It also enhances patient safety compared to intermittent monitoring.
- Wireless technology for continuously monitoring vital signs affects nursing care. It consumes time during day shifts and saves time during evening and night shifts.
- Contrary to expectations, nurses do not mention less contact with patients. These findings challenge thinking about if and how wireless technology for continuously monitoring of vital signs optimises fundamental nursing care delivery, which could influence nurse-sensitive outcomes and ‘care left undone’.
